# Lung Point Sign in Ultrasound Diagnostics of Pneumothorax: Imitations and Variants

**DOI:** 10.1155/2021/6897946

**Published:** 2021-05-28

**Authors:** Roman Skulec, Tomas Parizek, Martin David, Vojtech Matousek, Vladimir Cerny

**Affiliations:** ^1^Department of Anesthesiology, Perioperative Medicine and Intensive Care, J. E. Purkinje University, Masaryk Hospital Usti nad Labem, Socialni Pece 3316/12A, Usti nad Labem 400 11, Czech Republic; ^2^Teaching Department for Ultrasound Methods in Anesthesiology, Intensive Care and Emergency Medicine, Institute for Postgraduate Medical Education, Ruska 85, Prague 100 05, Czech Republic; ^3^Faculty of Health Studies, J. E. Purkinje University, Pasteurova 3544/1, Usti nad Labem 400 96, Czech Republic; ^4^Emergency Medical Service of the Central Bohemian Region, Vancurova 1544, Kladno 272 01, Czech Republic; ^5^Faculty of Medicine in Hradec Kralove, Charles University, Simkova 870, Hradec Kralove 500 03, Czech Republic; ^6^Usti and Labem Region Emergency Medical Services, Socialni Pece 799/7a, Usti nad Labem 400 11, Czech Republic; ^7^Department of Research and Development, Charles University in Prague, Faculty of Medicine in Hradec Kralove, University Hospital Hradec Kralove, Sokolska 581, Hradec Kralove 500 05, Czech Republic; ^8^Department of Anesthesia, Pain Management and Perioperative Medicine, Dalhousie University, Halifax, Nova Scotia, Canada

## Abstract

**Background:**

Pulmonary ultrasound plays a key role in the diagnosis of pneumothorax in emergency and intensive-care medicine. The lung point sign has been generally considered a pathognomonic diagnostic sign. Recently, several other situations have been published that can mimic the lung point, as well as a few different variants of the true lung point sign.

**Materials and Methods:**

Based on years of monitoring the literature and collecting our database of ultrasound findings, we prepared a review of ultrasound findings mimicking the lung point sign and ultrasound variants of the true lung point sign.

**Results:**

We present four imitations of the lung point sign (physiological lung point sign, pseudo-lung point sign, bleb point sign, and pleurofascial point sign) and two variants of the true lung point sign (double lung point sign and hydro point sign) documented by images and video records.

**Conclusions:**

Knowledge of ultrasound imitations and variants of the lung point sign may increase the reliability of pneumothorax diagnosis and may reduce the risk of performing unindicated interventions.

## 1. Introduction

Detection of pneumothorax is one of the typical examples of the effective use of bedside Point-of-Care Ultrasound (POCUS) in emergency medicine. Ultrasonographic diagnosis of pneumothorax was first described in veterinary medicine in horses and immediately implemented in human medicine [1, 2]. Since then, a number of studies have been published testing the reliability of the method, mostly against the gold standard, chest computed tomography [3]. Ding et al. published a meta-analysis of up to twenty studies and identified a sensitivity of 88 % and a specificity of 99 % for ultrasonographic diagnosis of pneumothorax [3].

One of the most reliable ultrasonographic findings in pneumothorax is a lung point sign. The lung point sign is an ultrasound image of the pleural interface of where a healthy lung starts and where the pneumothorax ends. Some authors have published up to 100 % reliability of this finding and described the finding as pathognomonic [4, 5]. This can lead to exaggerated expectations followed by inaccurate conclusions. In addition, several papers have been published in recent years that have highlighted ultrasonographic findings that mimic the lung point and may be the cause of diagnostic errors. In this review article, the authors summarize the literature and their own experience with findings mimicking the lung point sign and subject them to critical analysis.

## 2. Position of the Lung Point Sign in the Ultrasound Diagnosis of the Pneumothorax

**Figure 1:**
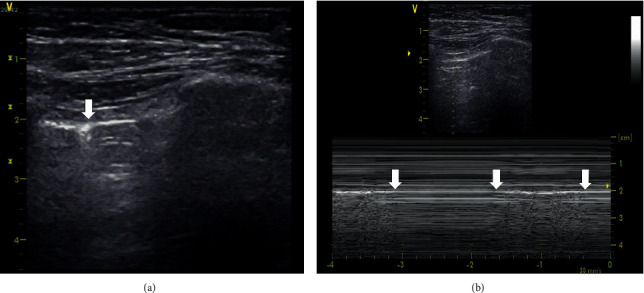
Lung point sign. (a) B-mode, linear probe. The vertical arrow indicates the lung point. On the left, pleural sliding is present and terminated by one Z line. On the right, pleural sliding is absent. (b) M-mode, linear probe. Arrows indicate the alternating seashore sign and barcode sign during respiration. A video loop is available as Supplementary File 1.

Ultrasonographic diagnosis of pneumothorax is based on a comprehensive assessment of several ultrasonographic signs in the context of the clinical setting. The ultrasonographic signs result from the pathophysiological nature of pneumothorax which is the loss of contact and mutual separation of the parietal and visceral pleura with the pleural space filled with air. This leads to the absence of pleural sliding, presence of a barcode sign (also referred to, in the literature, as a stratosphere sign), absence of lung pulse, absence of vertical artifacts, absence of the power slide phenomenon, and presence of the lung point sign (if the lung is not collapsed completely) [6]. The lung point sign is an ultrasonographic pleural phenomenon that occurs at the site where pneumothorax ends and normal contact between the parietal and visceral pleura is restored. When a probe is placed in intercostal space and a B-mode imaging is set, a pleural line without sliding into which pleural sliding regularly intervenes during respiration, often with vertical artifacts, can be found. In the M-mode, an alternating barcode sign and seashore sign are typically observed (Figure 1, Supplementary File 1) [4, 7]. The more laterally the lung point sign is identified, the greater the pneumothorax [8, 9].

The lung point sign is considered a pathognomonic sign of pneumothorax [4, 5]. It is associated with moderate sensitivity, about 66 % [4]. In principle, it cannot be observed in patients with pneumothorax with complete collapse of the lung and its detection may be difficult in all patients with diffuse attenuation of pleural sliding of etiology other than pneumothorax [6].

On the other hand, according to the original evidence, the presence of the lung point confirms, with 100 % certainty, the presence of pneumothorax [4, 5]. However, it has been published recently that there are several clinical situations that may simulate a lung point sign image but are not associated with the pneumothorax. In this article, the authors offer an overview of these situations and discuss the possibilities of distinguishing them from the real lung point sign in the pneumothorax.

## 3. Ultrasound Findings Mimicking the Lung Point Sign

### 3.1. Physiological Lung Point Sign

**Figure 2:**
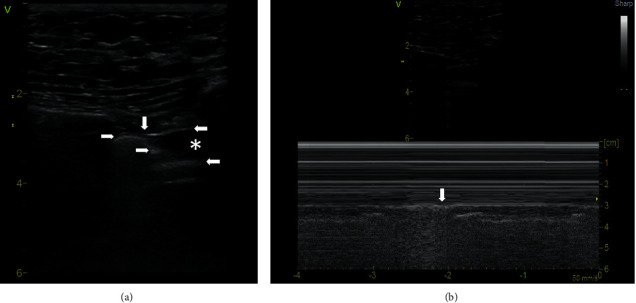
Physiological lung point. (a) B-mode, linear probe. The vertical arrow shows the physiological lung point. To the left of the vertical arrow, the upper horizontal arrow indicates pleural sliding, and to the right, the opposite horizontal arrow shows fascia endothoracica without pleural sliding. The lower horizontal left arrow indicates mediastinal pleura at the edge of the lung, and the lower horizontal right arrow points to the pericardium. The asterisks show mediastinal tissue. (b) M-mode, linear probe. The vertical arrow shows the physiological lung point. A video loop is available as Supplementary File 2.

Zhang and Chen introduced an imitation of the lung point sign and called it physiological lung point sign. They observed that applying the probe longitudinally at the 4th-5th intercostal space parasternally, it is possible to display, at the site of the mediastinal pleura, a tissue interface that is bounded by mediastinal tissue with the absence of pleural sliding and by a normally aerated lung with pleural sliding [10]. However, they do not specify a thin hyperechogenic line with the absence of sliding, which follows the pleural sliding. We are convinced that this is a fascia endothoracica. It is the layer of connective tissue which covers the inside of the chest wall and separates this from the underlying parietal pleura and other intrathoracic organs and mediastinal tissue. At the physiological lung point, where the lung ends, the mediastinal pleura penetrates to a depth and can usually be observed up to the pericardium which then continues horizontally. Together, these structures form a typical *Z*-shape (Figure 2, Supplementary File 2).

**Figure 3:**
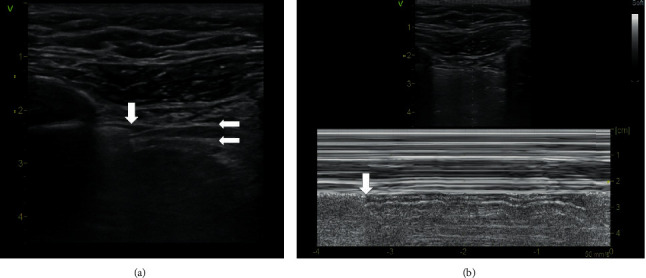
Physiological lung point sign with the pericardium adjacent to the fascia endothoracica. (a) B-mode, linear probe. The vertical arrow indicates the physiological lung point. The upper horizontal arrow shows the fascia endothoracica, and the arrow below points to the pericardium with a thin layer of mediastinal tissue between them. (b) M-mode, linear probe. The arrow indicates the physiological lung point. A video loop is available as Supplementary File 3.

The pericardium can be located at different depths, even just below the chest wall, but there is always mediastinal tissue between it and the fascia endothoracica (Figure 3, Supplementary File 3). The physiological lung point sign can be easily recognized from true lung point. In the area below the fascia endothoracica, beyond the edge of the lung, A lines are missing, a layer of mediastinal tissue may simulate a lung pulse, and a pulsating pericardium and myocardial wall appear in the image.

### 3.2. Pseudo-Lung Point Sign

**Figure 4:**
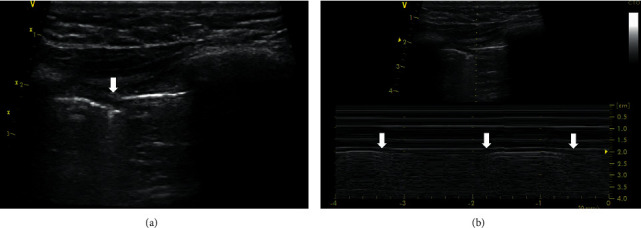
Pseudo-lung point sign. (a) B-mode, linear probe. The vertical arrow indicates the pseudo-lung point. On the left is the preserved pleural sliding, which is terminated by one lung comet. To the right of the arrow is the rigid contour of the pleura above the site of pulmonary contusion. In the pseudo-lung point site, pleural discontinuity is evident, which is not common in the pneumothorax. (b) M-mode, linear probe. Arrows indicate interfaces between a normally aerated and contused lung; however, no barcode sign is present. A video loop is available as Supplementary File 4.

The term pseudo-lung point sign was used by Gillman et al. to describe ultrasound finding of an obvious interface of sliding and absence of sliding at the pleural line over the site of pulmonary contusion without the presence of pneumothorax [11]. This phenomenon can be identified at the borderline of a contused and normally aerated lung. However, a fully expressed barcode sign is missing, lung pulse and B lines can be present, and interruption of the pleura may be observed (Figure 4, Supplementary File 4). On the other hand, the clinical condition of chest trauma with lung contusion is associated with a high probability of the presence of the pneumothorax, and this is associated with a higher risk of lung point misinterpretation.

### 3.3. Bleb Point Sign

**Figure 5:**
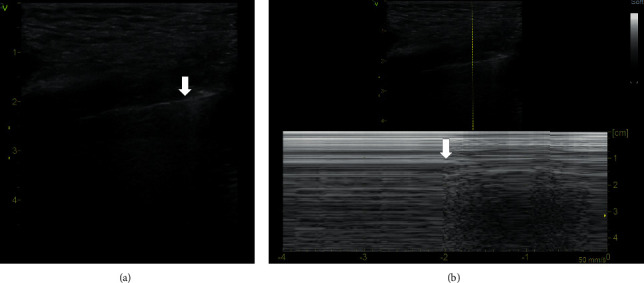
Bleb point sign. (a) B-mode, linear probe. The vertical arrow indicates the bleb point. Right to the arrow, pleural sliding is present and terminated by one lung comet. On the left, pleural sliding is absent. (b) M-mode, linear probe. Arrows indicate the bleb point. The barcode sign is present on the left, and the seashore sign is on the right. A video loop is available as Supplementary File 5.

In patients with bullous degeneration of the pulmonary parenchyma, the lung point sign may be observed at the border of the bulla and preserved aerated lung tissue. This finding does not differ from the lung point sign in pneumothorax (Figure 5, Supplementary File 5) [12–15]. In order for the bleb point sign phenomenon to occur, it is necessary for the lung tissue to completely disappear subpleurally at the bulla. Even a thin skin of the preserved lung tissue prevents the formation of this artifact [15].

Knowledge of this phenomenon is very important. The presence of a bleb point sign can result in a false positive diagnosis of pneumothorax and subsequent placement of a chest tube into the bulla. This is very dangerous and may require subsequent thoracic surgery. However, the problem is that unlike other imitations of the lung point sign, this one does not differ from the real lung point sign. This represents the real Achilles heel of ultrasonographic diagnostics of the pneumothorax. Therefore, when pneumothorax is suspected for any reason, it is always necessary to consider individually not only the ultrasound findings but also all other circumstances such as the clinical condition, the assumed probability of pneumothorax, the physical finding, medical history, and the results of other imaging techniques.

### 3.4. Curtain Sign and Pleurofascial Point Sign

**Figure 6:**
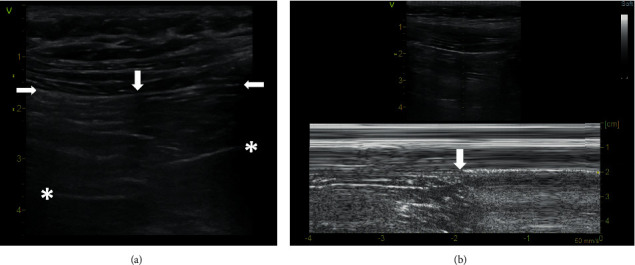
Curtain sign and pleurofascial point sign. (a) B-mode, linear probe. The vertical arrow indicates the pleurofascial point sign. To the left of it, the horizontal arrow shows the line of pleural sliding and the asterisk A line, and to the right of it, the horizontal arrow shows the fascia endothoracica and the asterisk indicates the peritoneum. (b) M-mode, linear probe. The arrow indicates the pleurofascial point. On the left is the record in the expiration, in the area of the fascia endothoracica, on the right is the seashore sign in the inspiration. A video loop is available as Supplementary File 6.

The curtain sign is the physiological ultrasound phenomenon regularly observed during examination of the basal pleura, lung bases, and diaphragm. It is generally used to describe cyclic appearance and disappearance of an aerated lung in the costophrenic recessus during respiration [16]. When applying the probe longitudinally at the coronal plane at the posterolateral alveolar and/or pleural syndrome point according to the BLUE protocol, the basal part of the lung dynamically enters the costophrenic recessus in the inspiration [17]. Thus, a line of pleural sliding appears with the usual A-profile in the scanned area, which veils the screen below. The line of pleural sliding is followed by a hyperechogenic line of fascia endothoracica. This does not exhibit the sliding phenomenon in ultrasound imaging. The interface between pleural sliding and fascia endothoracica mimics a lung point. Moreover, no vertical phenomena are present below the fascia. On the other hand, A-profile is absent, and a layer of the diaphragm is displayed below the fascia, followed by the peritoneum and the liver or spleen, according to the examined side. A stratospheric sign is missing in the M-mode, and the presence of a parenchymal organ subdiaphragmatically forms an image similar to a seashore sign (Figure 6, Supplementary File 6). Therefore, this physiological finding of the pleurofascial point sign should be easily distinguishable from the true lung point sign.

Ultrasonographic diagnosis of pneumothorax is based on the evaluation of more ultrasonographic signs than the lung point sign. A comparison of these signs with the findings in the situations associated with the imitation of the lung point sign is summarized in Table 1.

## 4. Less-Frequent Variants of the Lung Point Sign

### 4.1. Double Lung Point Sign

**Figure 7:**
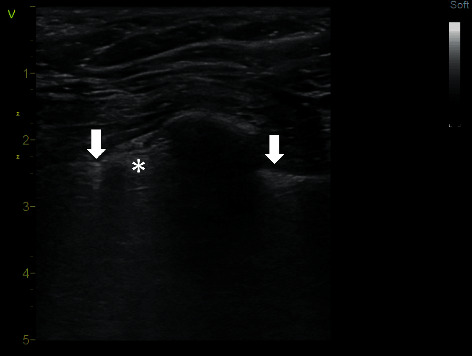
Double lung point sign. B-mode, linear probe. The left arrow shows the lung point on the left, which is bounded by the Z line, and the right arrow shows the pleural sliding reaching to the rib. Between the left arrow and the rib, a narrow section with the absence of sliding (marked by the asterisk) is visible, indicating a small strip of pneumothorax. A video loop is available as Supplementary File 7.

The double lung point sign is a powerful diagnostic finding for pneumothorax as well as the lung point sign [18–20]. It can be observed in very small pneumothorax, where the ultrasound beam is wide enough to cover both edges of the pneumothorax, and therefore, two lung points in one image can be identified. In the middle of the image is an area with the absence of sliding and on both sides, there is a visible pleural sliding, which appears in accordance with breathing (Figure 7, Supplementary File 7). The image of a double lung point sign may occur if pneumothorax is fixed between lung adhesions, may be due to blunt thoracic injury, but also can be observed as a complication of subclavian vein cannulation (our observation). Finding this sign requires a detailed examination of the entire hemithorax because it is a very discreet finding.

We must also emphasize that this double lung point sign related to pneumothorax has been described in adults. In newborns, the term “double lung point sign” has been used to describe an interface between relatively aerated superior lung fields and coalescent B lines in the basal lung fields in the patients with transient tachypnea of the newborn [21]. However, this observation is not related to pneumothorax and describes another pathophysiological situation.

## 5. Hydro Point Sign

**Figure 8:**
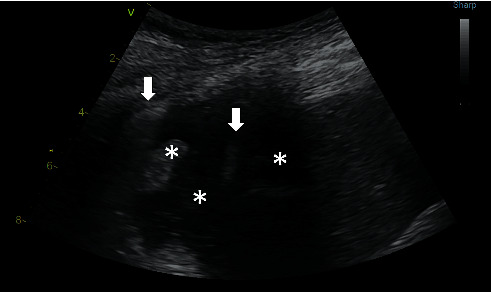
Hydro point sign. B-mode, a convex probe is placed longitudinally at the coronal plane in the posterior axillary line at the right lung base. The left arrow indicates a veiled pneumothorax artefact, and the right arrow shows a contour of the diaphragm. The collapsed lung is marked by the left asterisk, the middle asterisk indicates fluid in the pleural cavity, and the right asterisk shows an echogenic shadow of the rib, which partially covers the liver. A video loop is available as Supplementary File 8.

The hydro point sign is a specific finding characteristic for the presence of fluid and air in one pleural cavity [22]. This is probably a very reliable diagnostic finding because a unique combination of fluid and air in the pleural cavity is necessary for formation of this phenomenon. When scanning a patient in the semisitting position in the posterior axillary line, the probe is placed in the coronal plane with cranial orientation of the marker; if there is only fluid in the pleural cavity, it appears as an anechogenic or hypoechogenic zone and the diaphragm, chest wall, pleural space, and lung can be well identified. In the presence of fluidopneumothorax, if the ultrasound probe is at the level of the pleural fluid-air transition, the artefact of A-profile across the entire imaging field, such as a veil, enters the fluidothorax zone in inspiration and disappears in expiration (Figure 8, Supplementary File 8). Pleural sliding is absent, and a stratospheric sign is present.

## 6. Conclusions

Point-of-care ultrasound is a very reliable method of diagnosing pneumothorax, and the presence of the lung point sign is one of the most reliable ultrasonographic diagnostic features. In recent years, however, several findings have been described that mimic the lung point. These are not generated by the presence of pneumothorax, but by other conditions such as bullous degeneration of the pulmonary parenchyma, lung contusion, or various physiological tissue interfaces. Apart from the bleb point, few differences can be found in other ultrasonographic signs of pneumothorax, as described in this review. Therefore, in the diagnosis of pneumothorax, a comprehensive evaluation of all ultrasonographic signs is always necessary and must be interpreted in a clinical context, as is typical for the point-of-care approach.

## Figures and Tables

**Table 1:** A comparison of other ultrasonographic signs associated with the true lung point sign in pneumothorax with the signs related to the imitations of the lung point sign.

Ultrasound phenomenon	Interface of pleural sliding and absence of sliding	Barcode sign	A lines	Vertical phenomena	Lung pulse	Other specific findings
True lung point sign	+	+	+	−	−	−
Physiological lung point sign	+	−	−	−	+	Z-shape formed by the pleural line and pericardium; pulsating myocardial wall present
Pseudo-lung point sign	+	−	−	+	+	−
Bleb point sign	+	+	+	−	−	−
Pleurofascial point sign	+	−	−	−	−	Diaphragm and parenchymal organs below may be displayed

## Data Availability

The image ultrasound data used to support the findings of this study are included within the article as figures and within the supplementary information files as video loops.

## Supplementary Materials

Supplementary Materials All supplementary files are available under the link https://drive.google.com/drive/folders/13cIiAX0R97k2F3PyJ1GBVE2tn58MFm5G?usp=sharing. Supplementary File 1: a video loop demonstrating the lung point sign, associated with Figure 1. Supplementary File 2: a video loop demonstrating the physiological lung point sign, associated with Figure 2. Supplementary File 3: a video loop demonstrating the physiological lung point sign with the pericardium adjacent to the fascia endothoracica, associated with Figure 3. Supplementary File 4: a video loop demonstrating the pseudo-lung point sign, associated with Figure 4. Supplementary File 5: a video loop demonstrating the bleb point sign, associated with Figure 5. Supplementary File 6: a video loop demonstrating the pleurofascial point sign, associated with Figure 6. Supplementary File 7: a video loop demonstrating the double lung point sign, associated with Figure 7. Supplementary File 8: a video loop demonstrating the hydro point sign, associated with Figure 8.
