# Supplementary material for: Lung Point Sign in Ultrasound Diagnostics of Pneumothorax: Imitations and Variants
Source: Emerg Med Int. 2021 May 28;2021:6897946. doi: 10.1155/2021/6897946 (PMC8177981; doi:10.1155/2021/6897946)
Supplement: Supplementary Materials — All supplementary files are available under the link https://drive.google.com/drive/folders/13cIiAX0R97k2F3PyJ1GBVE2tn58MFm5G?usp=sharing. Supplementary File 1: a video loop demonstrating the lung point sign, associated with Figure 1. Supplementary File 2: a video loop demonstrating the physiological lung point sign, associated with Figure 2. Supplementary File 3: a video loop demonstrating the physiological lung point sign with the pericardium adjacent to the fascia endothoracica, associated with Figure 3. Supplementary File 4: a video loop demonstrating the pseudo-lung point sign, associated with Figure 4. Supplementary File 5: a video loop demonstrating the bleb point sign, associated with Figure 5. Supplementary File 6: a video loop demonstrating the pleurofascial point sign, associated with Figure 6. Supplementary File 7: a video loop demonstrating the double lung point sign, associated with Figure 7. Supplementary File 8: a video loop demonstrating the hydro point sign, associated with Figure 8. [file 6897946.f1.docx]

**SupplementaryMaterial**

All supplementaryfiles are availableunder the link https://drive.google.com/drive/folders/13cIiAX0R97k2F3PyJ1GBVE2tn58MFm5G?usp=sharing

Supplementary file 1

Video loop demonstrating the lung point sign. Associated with figure 1.

Supplementary file 2

Video loop demonstrating the physiological lung point sign. Associated with figure 2.

Supplementary file 3

Video loop demonstrating the physiological lung point sign with pericardium adjacent to the fascia endothoracica. Associated with figure 3.

Supplementary file 4

Video loop demonstrating the pseudo-lung point sign. Associated with figure 4.

Supplementary file 5

Video loop demonstrating the bleb point sign. Associated with figure 5.

Supplementary file 6

Video loop demonstrating the pleuro-fascial point sign. Associated with figure 6.

Supplementary file 7

Video loop demonstrating the double lung point sign. Associated with figure 7.

Supplementary file 8

Video loop demonstrating the hydro point sign. Associated with figure 8.
